# Prevalence and determinants of alcohol use disorder and its association with adherence to antihypertensive therapy among adult hypertensive patients in a hospital setting, Northwest Ethiopia

**DOI:** 10.1080/07853890.2026.2699553

**Published:** 2026-07-07

**Authors:** Gizachew Kassahun Bizuneh

**Affiliations:** Department of Pharmacognosy, School of Pharmacy, College of Medicine and Health Sciences, University of Gondar, Gondar, Ethiopia

**Keywords:** Alcohol use disorder, medication adherence, hypertension, antihypertensive therapy, Ethiopia

## Abstract

Background

Alcohol use disorder (AUD) is a major public health problem that contributes to poor treatment adherence and adverse clinical outcomes. Evidence on its impact on adherence to antihypertensive therapy remains limited in low-resource settings, including Ethiopia. This study assessed the prevalence of AUD and its association with adherence to antihypertensive medication among hypertensive adults.

**Methods:**

A hospital-based cross-sectional study was conducted among hypertensive outpatients at the University of Gondar Comprehensive and Specialized Hospital from January 30 to May 30, 2024. Participants were selected using simple random sampling. Alcohol use disorder was assessed using the Alcohol Use Disorders Identification Test (AUDIT) and medication adherence was evaluated over a three-month period. Bivariable and multivariable logistic regression analyses identifed factors associated with AUD and non-adherence to antihypertensive therapy. Statistical significance was set at *p* < 0.05.

**Results:**

A total of 400 participants were included (response rate: 100%), with a mean age of 44.9 ± 12.5 years. The prevalence of AUD was 12.2%, comprising hazardous drinking (8.0%), harmful use (2.5%), and alcohol dependence (1.7%). Male sex (AOR = 3.60; 95% CI: 1.30–9.94), cigarette smoking (AOR = 8.56; 95% CI: 3.89–18.82), and comorbidities (AOR = 3.87; 95% CI: 1.75–8.56) were independently associated with AUD. Overall, 42.2% of participants were non-adherent to antihypertensive therapy. Alcohol dependence was associated with nearly fourfold higher odds of poor adherence compared with social drinking.

**Conclusion:**

AUD is common among hypertensive patients and is significantly associated with poor adherence to antihypertensive therapy. Integrating alcohol use screening and intervention into routine hypertension care may improve medication adherence and treatment outcomes.

## Introduction

Hypertension is significant global health challenge and a leading contributor to cardiovascular disease-related morbidity and mortality [1,2]. Globally, more than half of the hypertensive patients have systolic blood pressure (SBP) ≥ 140 and/or diastolic blood pressure (DBP) ≥ 90 mmHg [3]. The cornerstone of hypertension control is the consistent use of antihypertensive drugs [4]. Nonetheless, poor adherence to these medications is a major issue, leading to resistant hypertension, an increased risk of cardiovascular events, more frequent healthcare utilization, reduced quality of life, and a greater burden on healthcare systems [5,6]. Multiple elements can undermine adherence to antihypertensive therapy. These include patient-related factors (forgetfulness, lack of knowledge, psychological factors, fear of side effects), medication-related factors (complex drug regimens, fear of side effects, high costs), condition-related factors (asymptomatic nature of hypertension, comorbidities), and social and environmental factors (social support, lifestyle habits, cultural beliefs) [7–10]. Among these, alcohol intake is consistently recognized as a major factor contributing to poor medication adherence [11]. Alcohol can interfere with antihypertensive therapy through several pathways: Regular alcohol consumption impairs cognitive function, judgment, and decision-making [12]. It can also interact with antihypertensive medications, worsening side effects such as dizziness or fatigue, and amplifying impact like hypotension [13]. Additionally, heavy alcohol users may engage in other harmful behaviors, such as smoking or drug use, which further disrupt medication adherence due to conflicting lifestyle challenges [14]. Chronic alcohol use may also lead to vasoconstriction, vascular hyper-reactivity, increased cytoplasmic calcium accumulation in smooth muscle cells, and heightened peripheral vascular resistance [15]. Excessive alcohol consumption can lead to resistance to antihypertensive drug treatment and increase the risk of serious cardiovascular complications, including ischemic and hemorrhagic stroke, alcohol-related cardiomyopathy, arrhythmias, and sudden cardiac death [16,17]. Research has shown that the rate of AUD is higher among those with hypertension than in the general population [18]. The prevalence of AUD among hypertensive patients varies by region: In Europe, AUD prevalence among hypertensive patients ranges from 15% to 30% [19]; in sub-Saharan Africa, Nigeria 24.1% [20], South Africa 18.5% [21], Tanzania 28.2% [22]; in Asia, India 19.3% [23], Pakistan 22.4% [24]; and in Latin America, Brazil 26.1% [25]. In Ethiopia, reported rates include 62.4% in Jimma Town, 21% in the Gurage Zone, 16% in Buta Jira, and 7.8% at Jimma University Hospital [26–29].

Previous studies have identified several determinants of alcohol use disorder (AUD) among patients with hypertension. Male sex has consistently been reported as a strong predictor of AUD, with odds ratios ranging from 2.3 to 3.1 globally, and similar findings reported in Ethiopia (AOR = 2.6) [30]. Current cigarette smoking is also significantly associated with AUD, with reported odds ratios between 1.8 and 2.5 [31]. In addition, low socioeconomic status has been linked to increased vulnerability to problematic alcohol consumption. The presence of comorbid conditions, particularly diabetes mellitus, has also been identified as an important determinant (OR ≈1.7), as demonstrated in African meta-analyses. These findings suggest that both behavioral and clinical factors contribute substantially to the risk of AUD among hypertensive patients. Despite widespread alcohol consumption in Ethiopia, there is limited data on how AUD affects adherence to antihypertensive therapy in this context. This study seeks to determine the prevalence of AUD and its impact on antihypertensive medication adherence among patients attending follow-up visits at UOGCSH. Screening for AUD in this population is crucial, as managing alcohol use effectively may improve adherence, enhance blood pressure control, and promote better health outcomes overall.

## Methods

### Study setting and period

This cross-sectional study was conducted at the outpatient department of the University of Gondar Comprehensive and Specialized Hospital (UOGCSH) from January 30 to May 30, 2024. Gondar is a historic city located 725 km northwest of Addis Ababa. The hospital serves as a referral center for approximately 6,800 hypertensive patients.

### Study population

Adults aged ≥18 years with a confirmed diagnosis of hypertension (SBP ≥140 mmHg or DBP ≥90 mmHg), receiving antihypertensive treatment for at least three months, and willing to provide informed consent were included.

### Sample size and sampling

A single population proportion formula was used, assuming 95% confidence, 5% margin of error, and 50% prevalence, yielding a sample size of 384. After applying a finite population correction, the final sample size was 400. Participants were selected using simple random sampling.

### Inclusion criteria and exclusion criteria

Adults (≥18 years), receiving antihypertensive treatment for a minimum of three months, with confirmed hypertension (SBP ≥140 mm Hg or DBP ≥90 mm Hg), and who are willing to provide informed consent were included in the study. Participants who refused to provide informed consent, were pregnant, had dementia or altered mentation, or were critically ill at the time of data collection were excluded from the study.

## Measurements

### Alcohol use disorders

Alcohol use disorder (AUD) was assessed using the Alcohol Use Disorders Identification Test (AUDIT), a 10-item screening tool originally developed by the World Health Organization (WHO) to identify patterns of hazardous and harmful drinking over the past 12 months. Each item is scored on a 0–4 scale, producing a total score between 0 and 40. Consistent with prior research, including our previously published study [32,33], a score of ≥8 was used to classify individuals as having probable AUD. Subcategories based on total score included: 1–7 (social drinking), 8–15 (hazardous drinking), 16–19 (harmful drinking), and ≥20 (probable alcohol dependence). AUDIT has demonstrated strong reliability and validity across diverse populations and settings, including primary care and community-based studies [34,35].

### Assessment of medication adherence

Medication adherence in this study was assessed using pill counts and medical chart refill records covering the preceding 90-day period. Patients were asked to bring their antihypertensive medications to the clinic visit, and the number of remaining tablets was counted. The number of pills taken was calculated by subtracting the number of remaining tablets from the number originally dispensed. Adherence percentage was then determined by dividing the number of pills taken by the number of pills prescribed for the 90-day period and multiplying by 100.

In addition, pharmacy refill information documented in the medical charts was reviewed to assess medication availability during the same 90-day period. The proportion of days covered (PDC) was calculated by dividing the number of days for which medication was available by 90 days and multiplying by 100. Patients were classified as adherent if they had access to their prescribed medications for ≥80% of days during the 90-day period and as non-adherent if medications were unavailable for ≥20% of days (i.e. ≥18 days without medication). This classification is consistent with previously published methods for assessing medication adherence using refill and self-report data [36].

### Study variables

The primary outcome variables of this study were the presence of alcohol use disorder (AUD) and non-adherence to antihypertensive medications. AUD was assessed using the World Health Organization’s Alcohol Use Disorders Identification Test (AUDIT) and categorized into social drinking, hazardous drinking, harmful drinking, and probable alcohol dependence. Antihypertensive medication non-adherence was defined as taking less than 80% of prescribed doses over the preceding three months. Independent variables included demographic factors such as age, gender, and marital status; clinical factors, including the presence of comorbidities, duration of hypertension, and number of antihypertensive medications; and behavioral factors, including substance use such as cigarette smoking and khat chewing, as well as dietary habits like salt consumption. This classification enabled the assessment of associations between AUD and antihypertensive non-adherence while accounting for potential demographic, clinical, and behavioral confounders.

### Definitions of terms

*Hypertension*: Defined as systolic blood pressure (SBP) ≥140 mmHg and/or diastolic blood pressure (DBP) ≥90 mmHg, or current use of antihypertensive medications [37].

*Antihypertensive medications*: Medications used for the management of hypertension, including angiotensin-converting enzyme inhibitors (ACEIs), angiotensin receptor blockers (ARBs), calcium channel blockers (CCBs), β-blockers, and diuretics [38].

*Alcohol Use Disorder (AUD)*: Assessed using the Alcohol Use Disorders Identification Test (AUDIT), with a score ≥8 indicating hazardous alcohol use [8–12], harmful use [13–19], and alcohol dependence (≥20), according to WHO criteria [32].

*Comorbidities*: Presence of at least one chronic coexisting medical condition, including diabetes mellitus, chronic kidney disease (CKD), coronary heart disease (CHD), and asthma or chronic obstructive pulmonary disease (COPD) [39].

### Data collection and data quality assurance

Data were collected using a structured questionnaire adopted from the previously published studies [24–27]. Data were collected by trained personnel using interviewer-administered questionnaires during routine outpatient visits at the University of Gondar Comprehensive Specialized Hospital (UOGCSH). Written informed consent was obtained from all participants prior to enrollment. Prior to conducting the main study, a pilot test of the questionnaire was conducted among a subset of hypertensive patients to ensure clarity, cultural relevance, and suitability for the study population. The instrument was translated into Amharic to ensure clarity, cultural relevance, and comprehension. To maintain data quality, completed forms were checked daily for completeness and consistency. Each participant was assigned a unique identification code, and all questionnaires were securely stored to ensure confidentiality.

### Data analysis

Continuous variables were summarized using means and standard deviations, while categorical variables were presented as frequencies and percentages. Binary logistic regression was used to determine the associations between predictor variables and outcome variables and those associated variables with P-value < 0.25 in the bivariable analysis were further analyzed using multivariable analysis to control for potential confounding factors. All statistical analyses were performed using SPSS version 26, with significance set at *p* < 0.05.

### Sensitivity analysis

To address potential prevalent user bias (healthy survivor bias), A sensitivity analysis was conducted restricted to patients who had initiated antihypertensive therapy within the past 24 months. The primary multivariable logistic regression model was repeated in this subgroup using the same covariate adjustments to evaluate whether the association between alcohol use disorder and medication non-adherence remained consistent.

## Results

### Sociodemographic and clinical characteristics

A total of 400 hypertensive patients participated in the study, with a complete response rate of 100%. The mean age was 44.9 years (SD = 12.5), and 58% were male. Most participants (67.5%) resided in urban areas, and 57% were married. The majority (78.3%) identified as Orthodox Christian. Regarding behavioral factors, 20% reported smoking cigarettes, 13.5% chewed khat, and 23.3% regularly consumed salt. Over one-third (35%) of participants had at least one comorbid condition, and nearly one-third had been living with hypertension for 5 to <10 years. The number of daily antihypertensive medications varied, with 32.8% taking one pill, 38.3% taking two pills, 19.8% taking three pills, and 9.3% taking four or more pills (Table 1).

**Table 1.** Sociodemographic and clinical characteristics of hypertensive patients at UOGCSH, 2024 (*n* = 400).

Variable	Category	Frequency (%)
Gender	Male	232 (58.0)
	Female	168 (42.0)
Age (years)	20–36	113 (28.3)
	38–44	118 (29.5)
	45–53	89 (22.2)
	54–75	80 (20.0)
Residence	Urban	270 (67.5)
	Rural	130 (32.5)
Marital status	Single	84 (21.0)
	Married	228 (57.0)
	Widowed	67 (16.8)
	Divorced	21 (5.3)
Religion	Orthodox	313 (78.3)
	Muslim	81 (20.3)
	Catholic	6 (1.5)
Living status	Alone	152 (38.0)
	With family	182 (45.5)
	With relatives	44 (11.0)
	With non-relatives	22 (5.5)
Education level	Unable to read and write	93 (23.3)
	Primary [1–8]	106 (26.5)
	Secondary [9–12]	92 (23.0)
	College and above	109 (27.3)
Employment	Not employed	124 (31.0)
	Private	176 (44)
	Government	100 (25)
Monthly income	Less than 1000	79 (19.8)
	1000–5000	105 (26.2)
	Greater than 5000	216 (54.0)
Smoking status	Yes	57 (14.2)
	No	343 (85.8)
Khat use	Yes	54 (13.5)
	No	346 (86.5)
Salt consumption	Yes	93 (23.3)
	No	306 (76.7)
Years lived with hypertension	<3 years.	109 (27.2)
	3 years <5 Yrs.	111 (27.8)
	5years to <10 Yrs.	124 (31.0)
	10 years and above	56 (12)
Comorbidity	Yes	140 (35.0)
	No	260 (65.0)
Number of pills per day	One pill	131 (32.8)
	Two pill	153 (38.3)
	Three pill	39 (19.8)
	Four and above pills	37 (9.3)
Duration since start of HTN drug(year)	6-12months	125 (31.3)
	13-24months	148 (37)
	> 24 months	127 (31.7)

### Sensitivity analysis

A sensitivity analysis was conducted restricting the sample to patients who had been on antihypertensive therapy for ≤12 months (*n* = 127) to approximate a new-user cohort. In this subgroup, alcohol dependence remained significantly associated with non-adherence to antihypertensive medication (AOR = 4.12, 95% CI: 1.45–11.67, *p* = 0.003).

### Prevalence of alcohol use and adherence

Among the study participants, 87.8% were classified as social drinkers (AUDIT score 1–7). The overall prevalence of alcohol use disorder (AUD) was 12.2%, comprising hazardous drinking (8%), harmful drinking (2.5%), and alcohol dependence (1.7%). Non-adherence to antihypertensive therapy was observed in 42.2% of participants (Table 2).

**Table 2.** Prevalence of alcohol use and antihypertensive therapy adherence (*n* = 400).

Characteristic	Category	Participants (N, %)
Alcohol use	Social drinkers	351 (87.8)
	Hazardous drinkers	32 (8)
	Harmful drinkers	10 (2.5)
	Dependence	7 (1.7)
Therapy adherence	Adherent	235 (58.8)
	Non-adherent	165 (42.2)

### Reasons for alcohol use

**Figure 1.**
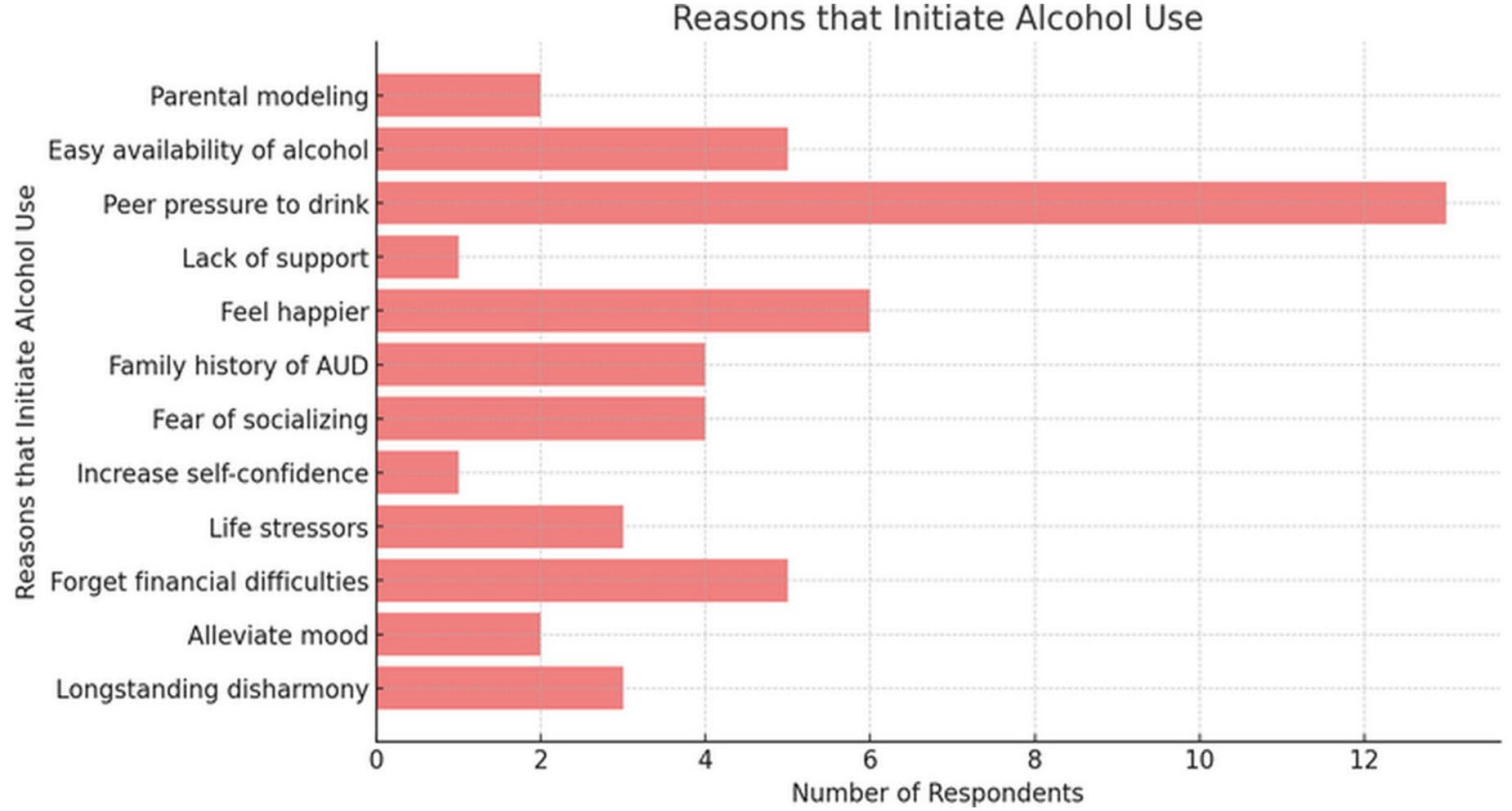
Reasons for alcohol use among hypertensive patients on follow-up at UOGCSH, 2024 (*n* = 400).

Among participants who consumed alcohol, 26.5% reported peer influence as the primary reason for drinking, followed by seeking happiness (12.2%), easy access to alcohol (10.2%), and coping with financial stress (10.2%) (Figure 1).

### Factors associated with alcohol use disorder

In multivariate analysis, being male was significantly associated with alcohol use disorder, with men having more than threefold higher odds compared to women (AOR = 3.60; 95% CI: 1.30–9.94). Current smoking was also strongly associated with AUD, with smokers having over eightfold higher odds than non-smokers (AOR = 8.56; 95% CI: 3.89–18.82). The presence of comorbid conditions significantly increased the likelihood of AUD, with affected patients having nearly fourfold higher odds (AOR = 3.87; 95% CI: 1.75–8.56). Age and educational status were not independently associated with AUD after adjustment. Regarding marital status, unmarried participants (single, widowed, or divorced) had lower odds of AUD compared to married participants (AOR = 0.38; 95% CI: 0.17–0.85). These findings highlight male sex, smoking, and comorbidities as key factors associated with alcohol use disorder among adult hypertensive patients at University of Gondar Hospital (Table 3).

**Table 3.** Factors associated with alcohol use disorder among adult hypertensive patients at university of gondar Hospital.

Variable	Category	AUD (n)	No AUD (n)	Crude OR (95% CI)	p-value	Adjusted OR (95% CI)	p-value
Sex	Male	39	193	3.19 (1.55–6.60)	0.002	3.60 (1.30–9.94)	0.013
	Female	10	158	1 (Reference)	–	1 (Reference)	–
Age (years)	20–36	9	104	1.29 (0.44–3.81)	0.645	1.09 (0.34–3.53)	0.890
	38–44	23	95	2.80 (1.23–6.35)	0.014	3.18 (1.21–8.33)	0.019
	45–53	12	77	2.12 (0.71–6.34)	0.175	2.07 (0.64–6.65)	0.224
	54–75	5	75	1 (Reference)	–	1 (Reference)	–
Residence	Urban	29	241	0.66 (0.36–1.22)	0.187	0.86 (0.41–1.84)	0.705
	Rural	20	110	1 (Reference)	–	1 (Reference)	–
Marital status	Married	23	205	1 (Reference)	–	1 (Reference)	–
	Unmarried*	26	146	0.46 (0.25–0.85)	0.013	0.38 (0.17–0.85)	0.019
Educational status	Unable to read/write	11	82	1.69 (0.65–4.41)	0.280	0.97 (0.24–3.98)	0.962
	Primary [1–8]	15	91	2.08 (0.84–5.14)	0.112	1.40 (0.40–4.90)	0.602
	Secondary [9–12]	15	77	2.46 (0.99–6.10)	0.052	2.45 (0.71–8.44)	0.157
	College and above	8	101	1 (Reference)	–	1 (Reference)	–
Employment status	Not employed	21	118	1.74 (0.76–3.98)	0.190	3.19 (0.96–10.60)	0.059
	Private	19	145	1.28 (0.56–2.96)	0.561	1.73 (0.51–5.85)	0.377
	Government	9	88	1 (Reference)	–	1 (Reference)	–
Smoking status	Yes	26	54	6.22 (3.31–11.69)	<0.001	8.56 (3.89–18.82)	<0.001
	No	23	297	1 (Reference)	–	1 (Reference)	–
Duration on anti-HTN drugs	6–12 months	18	107	1.02 (0.50–2.06)	0.959	1.22 (0.52–2.86)	0.645
	13–24 months	13	135	0.58 (0.27–1.24)	0.162	0.55 (0.22–1.39)	0.209
	>24 months	18	109	1 (Reference)	–	1 (Reference)	–
Comorbidity	Yes	26	118	2.23 (1.22–4.08)	0.009	3.87 (1.75–8.56)	0.001
	No	23	233	1 (Reference)	–	1 (Reference)	–

COR: crude odds ratio; AOR: adjusted odds ratio; CI: confidence interval; HTN: hypertension. Reference category = 1. *Unmarried = single, widowed, divorced.

### Factors associated with antihypertensive therapy adherence

At-risk drinking was associated with approximately a twofold increase in the likelihood of non-adherence to antihypertensive therapy (AOR = 1.83; 95% CI: 0.63–5.34), although this association was not statistically significant. In contrast, alcohol dependence was associated with nearly fourfold higher odds of non-adherence (AOR = 3.67; 95% CI: 1.27–10.64). Comorbidity was also significantly associated with non-adherence, with affected patients having approximately twofold higher odds of poor adherence (AOR = 2.07; 95% CI: 1.14–3.78). Other factors, including male sex (AOR = 1.67; 95% CI: 1.03–2.73), urban residence (AOR = 2.24; 95% CI: 1.34–3.75), lower educational status (unable to read/write: AOR = 2.20; 95% CI: 1.14–4.24; primary education: AOR = 2.06; 95% CI: 1.03–4.14), and higher number of daily pills (two pills: AOR = 4.95; 95% CI: 2.73–8.97; three pills: AOR = 5.60; 95% CI: 2.82–11.13; four or more pills: AOR = 6.62; 95% CI: 2.77–15.81), were also significantly associated with non-adherence (Table 4).

**Table 4.** Factors associated with antihypertensive therapy adherence among adult hypertensive patients at university of Gondar Hospital.

Variable	Category	Adherent (n)	Non-adherent (n)	Crude OR (95% CI)	p-value	Adjusted OR (95% CI)	p-value
Sex	Male	129	103	1.50 (0.99–2.24)	0.056	1.67 (1.03–2.73)	0.039
	Female	106	62	1 (Reference)	–	1 (Reference)	–
Age (years)	20–36	72	41	0.95 (0.52–1.72)	0.860	1.02 (0.51–2.03)	0.967
	38–44	68	50	1.23 (0.69–2.19)	0.490	1.10 (0.56–2.18)	0.785
	45–53	45	44	1.63 (0.88–3.01)	0.120	1.59 (0.77–3.25)	0.207
	54–75	50	30	1 (Reference)	–	1 (Reference)	–
Residence	Urban	144	126	2.04 (1.31–3.19)	0.002	2.24 (1.34–3.75)	0.002
	Rural	91	39	1 (Reference)	–	1 (Reference)	–
Educational status	Unable to read/write	70	56	1.63 (0.93–2.83)	0.086	2.20 (1.14–4.24)	0.018
	Primary [1–8]	48	41	1.74 (0.95–3.16)	0.071	2.06 (1.03–4.14)	0.042
	Secondary [9–12]	54	37	1.39 (0.76–2.54)	0.279	1.43 (0.70–2.90)	0.324
	College and above	63	31	1 (Reference)	–	1 (Reference)	–
Duration on HTN drugs	6–12 months	83	42	0.62 (0.37–1.04)	0.070	0.59 (0.33–1.06)	0.077
	13–24 months	82	66	0.99 (0.61–1.59)	0.960	0.90 (0.52–1.56)	0.705
	>24 months	70	57	1 (Reference)	–	1 (Reference)	–
Comorbidity	Yes	55	54	1.59 (1.02–2.48)	0.040	2.07 (1.14–3.78)	0.017
	No	180	111	1 (Reference)	–	1 (Reference)	–
Monthly income	<1000 birr	63	64	1.66 (1.04–2.65)	0.030	1.53 (0.85–2.75)	0.155
	1000–5000 birr	69	38	0.90 (0.54–1.49)	0.680	1.24 (0.68–2.28)	0.484
	>5000 birr	103	63	1 (Reference)	–	1 (Reference)	–
Number of pills	One pill	107	24	1 (Reference)	–	1 (Reference)	–
	Two pills	75	78	4.64 (2.69–7.99)	<0.001	4.95 (2.73–8.97)	<0.001
	Three pills	38	41	4.81 (2.58–8.99)	<0.001	5.60 (2.82–11.13)	<0.001
	Four & above pills	15	22	6.54 (2.96–14.43)	<0.001	6.62 (2.77–15.81)	<0.001
Alcohol use disorder	Social drinking	215	132	1 (Reference)	–	1 (Reference)	–
	At-risk drinking*	12	11	1.80 (0.66–4.87)	0.249	1.83 (0.63–5.34)	0.270
	Dependence	8	22	4.48 (1.94–10.35)	<0.001	3.67 (1.27–10.64)	0.017

COR: crude odds ratio; AOR: adjusted odds ratio; CI: confidence interval; HTN: hypertension. Reference category = 1.

*At-risk drinking = combined hazardous + harmful drinking (AUDIT-based categories with <5 cases individually).

## Discussion

Alcohol use disorder (AUD) ranks among the most widespread psychiatric conditions worldwide, affecting a substantial number of people [40]. Globally, approximately 2.3 billion people consume alcohol, with over 60% engaging in heavy episodic drinking [41]. Alcohol contributes to 5.3% of total mortality and 5.1% of the global burden of disease, and it is associated with medication non-adherence, substance use, suicide, homicide, decreased productivity, and multiple physical comorbidities [32–44]. Despite its significance, limited data exist regarding the prevalence and impact of AUD on antihypertensive therapy adherence in resource-limited settings like Ethiopia. This study sought to address this knowledge gap by assessing the prevalence of AUD and its association with medication adherence among hypertensive patients at the University of Gondar Comprehensive Specialized Hospital.

Our investigation identified a 12.2% prevalence of AUD among hypertensive patients, a figure that is comparatively lower than several community-based Ethiopian studies, where rates ranged from 16% to over 60%. This variation may be attributed to differences in study populations, as our cohort mainly included older adults under continuous medical supervision, which may discourage frequent or heavy drinking. Notably, our result is marginally higher than the 7.8% prevalence previously reported in a hospital-based study from Jimma.

Globally, alcohol misuse is prevalent and is recognized as a primary contributor to a range of chronic diseases, including liver disease and an important risk factor for cancers of the esophagus, larynx, mouth, liver, and breast cancer [45,46]. Importantly, elevated alcohol intake has been consistently linked to increased blood pressure [47,48] positioning it as a significant modifiable risk factor in the development and progression of hypertension [49,50].

Consistent with previous literature, comorbidities male sex, cigarette smoking, and were significantly associated with AUD. The current study revealed that comorbidity is significantly associated with alcohol use disorder, aligning with findings by Sterling et al. (2020) [51], which showed that patients with certain medical conditions were more likely to have elevated levels of alcohol use.

The present study, found that males were 3.60 times more likely than females to have Alcohol Use Disorder (AUD), aligning with similar findings from studies in Singapore [52] and the United States [53], which have reported higher AUD prevalence, alcohol-related harms, and alcohol-related fatalities among men [54–56].

Furthermore, our study revealed a statistically significant correlation between AUD and cigarette smoking. This finding is consistent with other studies indicating that smokers have a higher likelihood of developing AUD compared to non-smokers, and individuals with AUD are more likely to smoke cigarettes [57]. This correlation may be attributed to nicotine’s ability to activate certain neural pathways that can increase alcohol cravings. The co-occurrence of smoking and AUD poses additional health risks, heightening the likelihood of chronic diseases such as cardiovascular issues, respiratory problems, and certain cancers [58,59].

Non-adherence to antihypertensive therapy was observed in 42.2% of participants, consistent with prior studies showing suboptimal adherence in similar populations. Alcohol consumption significantly impacts therapy adherence among individuals with hypertension. This aligns with findings from Grodensky et al. [11], who reported a strong association between alcohol misuse and poor adherence, with alcohol drinkers being more likely nonadherent to antihypertensive medication compared to nondrinkers. Consistent with this, our findings show that at-risk drinking (combining hazardous and harmful drinking) was not significantly associated with non-adherence (AOR = 1.83; 95% CI: 0.63–5.34), whereas alcohol dependence was significantly associated with nearly fourfold higher odds of non-adherence (AOR = 3.67; 95% CI: 1.27–10.64), indicating a progressively stronger association between the severity of alcohol use and reduced adherence to antihypertensive therapy. These results suggest that alcohol use is a major factor of non-adherence, with alcohol-dependent individuals being nearly four times more likely to exhibit low adherence compared to social drinkers. The present findings revealed that alcohol use is the key factor for medication nonadherence in hypertensive patients. Beyond alcohol dependence, several other factors were significantly associated with poor adherence to antihypertensive therapy in our study. Male sex was linked to higher odds of non-adherence, consistent with previous findings that men are often less likely to engage in long-term treatment regimens due to lower health-seeking behaviors [60,61]. Urban residence was also associated with non-adherence, which may reflect lifestyle factors, higher stress levels, or greater reliance on self-medication in urban settings [62,63].

Lower educational status, particularly being unable to read/write or having only primary education, was another significant determinant, highlighting the role of health literacy in understanding treatment instructions and the importance of patient education [64,65]. Higher pill burden emerged as a strong predictor of non-adherence; participants taking two or more antihypertensive medications daily had progressively higher odds of poor adherence, emphasizing the challenges of polypharmacy [66,67].

Finally, comorbid conditions increased the likelihood of non-adherence, possibly due to competing health priorities, complex medication schedules, or heightened risk of side effects [68,69]. These findings indicate that non-adherence to antihypertensive therapy is multifactorial, involving socio-demographic, clinical, and treatment-related determinants, and that interventions should be tailored to address these diverse contributing factors.

To address potential prevalent user bias, a sensitivity analysis was performed restricted to patients with ≤12 months of antihypertensive treatment, approximating a new-user cohort. The association between alcohol dependence and medication non-adherence remained statistically significant and slightly stronger in this subgroup. This finding suggests that the observed relationship is robust and unlikely to be explained by survivor bias among long-term medication users. Nevertheless, due to the cross-sectional design, residual bias cannot be completely excluded.

### Strengths and limitations of the study

This study provides valuable evidence on the prevalence of alcohol use disorder and its association with adherence to antihypertensive medication in a resource-limited setting. The use of a validated screening tool (AUDIT) and a robust multivariable analytical approach strengthens the reliability of the findings. However, the cross-sectional design precludes causal inference, and reliance on self-reported data may introduce recall and social desirability bias. Although a sensitivity analysis restricted to patients with shorter treatment duration was performed to approximate a new-user cohort, the cross-sectional nature of the study limits the ability to fully eliminate potential prevalent user bias. Additionally, as this study was conducted at a single tertiary hospital in Northwest Ethiopia, the generalizability of the findings to other regions or healthcare settings may be limited. Variations in healthcare access, patient characteristics, and cultural practices could influence alcohol use and medication adherence. Therefore, future research involving multiple centers and more diverse populations, preferably using longitudinal or new-user cohort designs, would enhance external validity and provide more robust evidence regarding the relationship between alcohol use and adherence to antihypertensive therapy.

## Conclusion

This study demonstrates a substantial prevalence of alcohol use disorder among hypertensive patients, with male sex, cigarette smoking, and comorbidities identified as significant risk factors. AUD was strongly associated with poor adherence to antihypertensive therapy, emphasizing the need for integrated healthcare strategies that concurrently address alcohol use and hypertension management. Interventions targeting alcohol reduction could improve medication adherence, blood pressure control, and overall health outcomes in hypertensive populations. Future research should explore effective approaches to managing AUD in hypertensive patients and investigate the long-term impact of alcohol consumption on hypertension and related comorbidities.

## Data Availability

The data used in these findings are available in the manuscript and will be shared upon request from the corresponding author.
